# Theater-based interventions as a means of reducing social isolation and loneliness, facilitating successful aging, and strengthening social cognition in older adults

**DOI:** 10.3389/fpsyg.2024.1364509

**Published:** 2024-06-28

**Authors:** Brennan McDonald, Andrea M. F. Reiter, Philipp Kanske

**Affiliations:** ^1^Clinical Psychology and Behavioral Neuroscience, Faculty of Psychology, Technische Universität Dresden, Dresden, Saxony, Germany; ^2^German Center of Prevention Research on Mental Health, Würzburg, Bavaria, Germany; ^3^Department of Psychology, Faculty of Medicine, University of Würzburg, Würzburg, Germany; ^4^Clinic and Polyclinic for Child and Adolescent Psychiatry, Psychosomatics and Psychotherapy, University Hospital Würzburg, Würzburg, Bavaria, Germany

**Keywords:** social cognition, empathy, theory of mind, elderly, wellbeing, acting training, leisure activities

## Introduction

The distinct but related experiences of social isolation (the objective lack of social contact with others) and loneliness (a distressing affective state caused by the absence of social contact) can have profound, negative impacts on physical and mental wellbeing across all ages (Leigh-Hunt et al., 2017), rivaling well-established mortality risks such as smoking, high blood pressure, and obesity (Cacioppo and Cacioppo, 2018). Whilst social isolation and loneliness (SI/L) occur across the life span, older adults are a population noted to be especially vulnerable to these experiences, along with the associated negative impacts on mental health, physical health, and wellbeing (Anderson and Thayer, 2018; O'Súilleabháin et al., 2019; Cudjoe et al., 2020; World Health Organization, 2021). Given this vulnerability, developing interventions to ameliorate SI/L in older adults is of clear practical importance.

Interestingly, theater-based interventions have gained traction in recent years as a means of enhancing healthy cognitive aging by providing complex, dynamic, and cognitively demanding tasks (Noice et al., 2015; Noice and Noice, 2021). Building upon these findings, this article proposes that theater-based interventions may also provide complex and varied *social tasks* to older adults, with the potential to reduce SI/L by encouraging frequent and engaging social interactions, strengthening social cognition, and facilitating successful aging.

## Prevalence of social isolation and loneliness in older adults and associated risk factors

Predictions from the World Health Organization indicate that the population of older adults (aged 60+) will significantly expand over the coming decades (United Nations, 2019). With this increase comes a corresponding rise in the physical and mental health challenges associated with older age. In particular, the prevalence of SI/L in older adults is of increasing global concern (World Health Organization, 2021), with SI/L being linked to age-related health deterioration, including an increased risk of premature death (Steptoe et al., 2013; Foster et al., 2023), cognitive decline (Lara et al., 2019; Freak-Poli et al., 2022), neurodegenerative disease (Huang et al., 2023), malnutrition (Boulos et al., 2017) cardiovascular disease and stroke (Valtorta et al., 2016; Vallée, 2023), along with higher rates of depression, anxiety, and suicide (National Academies of Sciences Engineering and Medicine, 2020; Zhang et al., 2023).

Cross-national epidemiological data indicate that a significant percentage of older adults are adversely impacted by SI/L, including findings from the UK (Foster et al., 2023), Australia (Ogrin et al., 2021), Malaysia (Ibrahim et al., 2013), Canada (de Jong Gierveld et al., 2015), the Netherlands (Holwerda et al., 2016), Germany (Beller and Wagner, 2018; Röhr et al., 2021), and the USA (Anderson and Thayer, 2018; Cudjoe et al., 2020; Malani et al., 2023). For example, Surkalim et al. (2022) investigated loneliness across 40 European countries, finding a prevalence in northern European countries of 4.2%−6.5% and in eastern European countries of 18.7%−24.2% for older adults. Further estimates from Gao et al. (2021) in older adults suggest a prevalence of loneliness of between 25.3 and 32.4% in Latin American countries, 18.3% in India and 3.8% in China. However, other results for the prevalence of loneliness in older adults were 44% in India (Hossain et al., 2020) and 29.6% in China (Yang and Victor, 2008), highlighting that the specific estimates may vary depending on the study population, age groups, and measurement tools. Regarding social isolation, in Germany a prevalence of 21.7% was observed in a large cohort of older adults (Röhr et al., 2021). In the USA, estimates similarly indicate that 24%−34% of the elderly population are impacted by social isolation (4% characterized as severely socially isolated) and 43% by loneliness (Anderson and Thayer, 2018; Cudjoe et al., 2020). This is further supported by a representative sample of older US adults in the University of Michigan National Poll on Healthy Aging (Malani et al., 2023), in which participants self-reported feelings of being isolated from others (29% some of the time, 5% often), a lack of companionship (29% some of the time, 8% often) and infrequent contact (once a week or less) with people from outside their home (14% once a week, 10% every 2–3 weeks, 9% once a month or less). Viewed broadly, recent global estimates encompassing 41 studies suggest a pooled prevalence of social isolation of 26% among community-dwelling older adults (Teo et al., 2023). This is consistent with the recent advocacy brief of the WHO, estimating the prevalence of SI/L in older adults at 20–34% in China, Europe, Latin America, and the USA (World Health Organization, 2021). The brief further identifies SI/L in older adults as a global priority public health problem, noting in particular that these concerns have been made more salient by the COVID-19 pandemic.

Regarding risk, physical (e.g., Philip et al., 2020), psychological (e.g., Aartsen and Jylhä, 2011), economic (e.g., Pikhartova et al., 2016; Dahlberg et al., 2022), work/family (e.g., Chen and Feeley, 2014; Dahlberg et al., 2022), environmental (e.g., Prieto-Flores et al., 2011), and demographic factors (e.g., Wu and Penning, 2015; Perone et al., 2020) have each been identified as contributing to SI/L in older adults (for reviews see Nicholson, 2012; Dahlberg et al., 2022). For example, commonly identified physical risk factors for social isolation include chronic disease, hearing loss, or reduced mobility (Tomaka et al., 2006; National Academies of Sciences Engineering and Medicine, 2020; Tomida et al., 2024), while demographic factors include membership in minority groups (e.g. Jopling, 2015; Wu and Penning, 2015; Perone et al., 2020) and differences between the genders (Umberson et al., 2022). Examples with regard to work and family include reduced social support after retirement (Chen and Feeley, 2014) and/or the death of close friends or loved ones (in particular one's spouse), with both frequently identified as significant risk factor to increased social isolation in older adults (Dahlberg et al., 2022). Environmental contributions such as limited access to digital technology, insufficient transportation, and living in remote areas are also identified risk factors (Holt-Lunstad, 2017; World Health Organization, 2021). Importantly, much the same risk factors for loneliness in older adults have also been identified, including losing a spouse, reduced social activities, increased physical disabilities, and poor mental health (Victor et al., 2005; Aartsen and Jylhä, 2011).

Given the global issue of SI/L in older adults, numerous public policy initiatives and charities have been created to combat these negative social experiences, including in the UK (Age UK, 2015), Denmark (Sagen, 2024), Australia (Ending Loneliness Together, 2024), Ireland (ALONE, 2024), the Meuse-Rhine Euroregion (euPrevent PROFILE project, 2023) and New Zealand (Age Concern New Zealand, 2024). For example, the *Campaign to End Loneliness* in the UK and *Ending Loneliness Together* in Australia were both established to facilitate social connections among older individuals, as well as develop interventions that highlight and address SI/L in older adults (Age UK, 2015; Ending Loneliness Together, 2024). Regarding the possible types of interventions, Cotterell et al. (2018) in their review of the topic highlight several different categories aimed at combating SI/L in older adults, including one-to-one interventions, group interventions, interventions focused on providing services, technology-based interventions, and community interventions. Such interventions can be conducted in a traditional clinical setting or be the outcome of policy initiatives in the wider community. For example, one-to-one interventions can include cognitive behavioral therapy and psychotherapy with a professional therapist (Jarvis et al., 2019; Smith et al., 2021), as well as community befriending schemes in which older adults are put in contact with volunteers with the purpose of facilitating friendships (Fakoya et al., 2021). Group interventions are designed to facilitate social interaction among older adults while pursuing activities based on shared interests. For example, exercise, choir, or dance groups have all been identified as potential means of reducing loneliness in older adults (Johnson et al., 2020; Hansen et al., 2021; Mays et al., 2021). Technology-based interventions have also gained increasing interest as a means of providing direct support for Internet and computer usage, assistance for video calls, messaging platforms, and online forums, as well as policy related to digital inclusion strategies for older adults (Shah et al., 2021).

Fundamentally, the principle goal of any such social intervention aimed at reducing SI/L in older adults is also to produce a corresponding measurable increase wellbeing, defined as how people evaluate the quality of their lives, drawing from their own experiences, contributions to their community, relationships, emotions, and overall functioning (Ruggeri et al., 2020). Importantly, the association between SI/L and negatively impacted wellbeing in older adults is well established (Leigh-Hunt et al., 2017; Anderson and Thayer, 2018; O'Súilleabháin et al., 2019; Cudjoe et al., 2020). However, it is also important to note that only limited evidence currently exists to support the effectiveness of any single, specific type of intervention, primarily due to the small number of studies for any given program (Cotterell et al., 2018; Gardiner et al., 2018; Fakoya et al., 2020; World Health Organization, 2021; Hoang et al., 2022). Still, when taken in together, the available evidence indicates that key features of successful interventions against SI/L in older adults involve a grounding in theoretical frameworks, a focus on prevention, providing social activities in a group setting, as well as encouraging active participation (Dickens et al., 2011; Cotterell et al., 2018).

## Leisure activities and successful aging

One feature of daily life that is strongly linked to positive wellbeing is frequent engagement in leisure activities (i.e. those activities people engage in during free time; Pressman et al., 2009; Adams et al., 2011; Mock and Smale, 2023). Such leisure activities can include for example voluntary work, sports teams, art or music groups, theater performance, or book clubs. Importantly, benefits of leisure activities are shown to be manifold, with such activities helping older adults maintain and improve cognitive, physical, and mental health (Nimrod, 2007; Chang et al., 2014; Li J. et al., 2021). In addition, leisure activities are often social in nature and have been shown to explain a significant part of older people's social connectedness (Toepoel, 2013). There is also a strong suggestion that leisure activities are able to reduce SI/L in older adults by encouraging social engagement (Glover, 2019; Hajek and König, 2019; Teh and Tey, 2019; Li W. et al., 2021). Based on these physical, cognitive, social, and mental health benefits, efforts have been made to develop leisure activities into intervention programs capable of producing replicable improvements in health and wellbeing (Adam-Castelló et al., 2023), including theater-based interventions (Pestana et al., 2020; Noice and Noice, 2021). Furthermore, given the importance of leisure activities for health and wellbeing in the elderly, such engagement appears to have a crucial role for successful aging (Sala et al., 2019).

The *successful aging model* (contrasted against *usual aging* which focuses solely on the absence of disease), encompasses optimal aging characterized by high cognitive and physical function, low likelihood of disease and disability, and active participation in life (Rowe and Kahn, 1997), with more recent conceptualizations expanded to include mental health dimensions such as positive wellbeing (Pruchno et al., 2010; Paggi et al., 2016). A corollary of this definition is that individuals who are successfully aging are less likely to suffer from SI/L. Indeed, an important component within successful aging is frequent social engagement (Mendes de Leon, 2005), with involvement in productive social activities deemed crucial for life satisfaction in older age (Neugarten et al., 1961; Kim and Park, 2024). For this reason, various authors have highlighted the association between engaging in leisure activities and successful aging (DeCarlo, 1974; Chaves et al., 2009; Lee and Payne, 2015). Given that theater performance is an established leisure activity (Michalos and Kahlke, 2008; Pestana et al., 2020), we suggest that theater-based interventions with older adults may offer a structured social leisure program with the inherent goal of fostering social connectedness, wellbeing, and ultimately successful aging in older adults.

## The potential of theater-based interventions to encourage social interaction, reduce social isolation and loneliness, and facilitate successful aging in older adults

In recent years, theater-based interventions have gained traction as a means of enhancing healthy cognitive aging. Pioneering work over the last 35 years by Helga and Tony Noice has demonstrated that theater-based interventions are capable of producing robust and replicable gains on numerous standard measures of cognitive functioning in older adults, including executive function, memory, language comprehension, problem solving, and creativity (for substantive reviews see, Noice et al., 2015; Noice and Noice, 2021). Building upon these findings, we propose here that along with enhancing healthy cognitive aging, theater-based interventions may also offer a potential means to encourage social interaction, reduce SI/L, and facilitate successful aging in older adults.

Broadly understood, theater-based interventions involve a group of participants coming together to undertake acting training and/or discuss, develop, and practice scripted or improvised dramas for the purpose of theatrical performance (either under the tutorage of a trained professional or as a self-directed group). Here, we understand theatrical performance as planed or spontaneous interactions between participants undertaking the goal of enacting characters in environments through pretense, embodiment, and play. Given this definition, there exists a wide variety of possible ways to implement theatrical performance as a social leisure program. As one such example, Banducci et al. (2017) investigated whether acting training improves episodic memory recall in older adults. The intervention implemented by the researchers involved two groups; an acting training group and an active control group. Over the course of 4 weeks, both the control and intervention sessions were held twice weekly, each lasting 75 min. Additionally, there was a 15-min coffee break incorporated into the sessions to promote additional social interaction among the participants. In the acting training group, participants engaged in brief scenes with partners using drama scripts, under the tutorage of professional trainers. The acting training activities aimed to teach several main concepts related to acting performance. Firstly, participants were advised against mere “pretending” and instead encouraged to enact actions realistically. Secondly, they were prompted to imagine and mentally construct the scenarios they were to act out. Thirdly, they were taught to be driven by the goals outlined in the scripts, working through challenges to achieve their character's objectives. In the first 2 weeks, memorization of scripts wasn't required, and spontaneity in responding to interactions or changes was emphasized. Throughout the training, participants were urged to engage with these concepts intellectually, emotionally, and physically. These principles were integrated into various lessons over the intervention period. In the final 2 weeks, participants were expected to perform their scenes verbatim from memory. In contrast, the active control group took part in a theater appreciation class covering acting styles and theater history through talks, demonstrations, and video clips, controlling for improvements solely from learning about acting and social interaction. Thus, the acting group undertook both acting training and engaged in theatrical performance throughout the course of the intervention, with additional features (i.e., a coffee break) to encourage social interactions and relationship building. For other representative examples of various theater-based interventions in practice see Noice et al. (1999), Noice et al. (2004), Noice and Noice (2008), Noice and Noice (2013), Yuen et al. (2011), and Schwenke et al. (2021).

A common aspect across these theater-based interventions is their inherent social nature, conducted in peer groups and thus providing a high degree of social interaction. As discussed in the previous section, social interaction is strongly associated with reduced SI/L, successful aging, and corresponding wellbeing. Indeed, a recent study found that younger adults in a 6-weeks performing arts training program reported higher levels of psychological wellbeing after completing the program (Schwenke et al., 2021). Similar findings for wellbeing were also observed in older adults participating in a theater program who specifically reported enjoying the social interaction facilitated by the intervention (Yuen et al., 2011). Theater-based interventions can thus be viewed at as social leisure programs that facilitates social engagement and, in doing so, successful aging. There is also broad evidence indicating that participation in theater-based interventions by older adults reduces age-related risk of Alzheimer's disease and other forms of dementia (Noice and Noice, 2018), contrasted against SI/L which increases the risk of all-cause dementia and Alzheimer's disease (Sundström et al., 2020; Shen et al., 2022; Shafighi et al., 2023). In addition, theater-based interventions are often also a new experience for participants, with novelty an important aspect of stimulating cognitive and social activities associated with health across the lifespan (Hultsch et al., 1999; Mather, 2020).

Finally, interventions that facilitate social interaction have been identified are playing an important role in mitigating SI/L (Gardiner et al., 2018). In addition, interventions are more effective at reducing SI/L if the older adults are involved in the planning, developing and execution of activities (Wylie, 2012; Bartlett et al., 2013), with productive and challenging tasks appearing more effective than passive activities (Howat et al., 2004; Pettigrew and Roberts, 2008). Indeed, in this respect, a principle benefit of theater-based interventions is that they offer complex, varied, fulfilling, and enjoyable activities involving embodied cognitive, social, and emotional engagement (Noice and Noice, 2021). Participants are able to be actively involved in creating the performance, which can be adapted to their requirements (e.g., mobility). Thus, when taken together, theater-based interventions provide frequent social leisure activities that offers a high degree of planning and control to older participants, while also supplying complex and varied social, cognitive, physical, and emotional tasks that are engaging and intrinsically rewarding. For these reasons, theater-based interventions are a promising candidate for future investigations into reducing SI/L, facilitating successful aging, and promoting wellbeing in older adults.

## Theater-based interventions may also strengthen social cognition in older adults

In this final section, we suggest that theater-based interventions may have the potential to strengthen social cognition in older adults. Social cognition is defined as the ability to use abstract, propositional reasoning (e.g., about thoughts, desires, or feelings) to represent others' mental processes (Happé et al., 2017). Importantly, this ability is an aspect of social behavior that plays a prominent role in successful social functioning (Frith and Frith, 2006) and maladaptive social cognition is associated with SI/L and connected harms (Cacioppo et al., 2015; Lieberz et al., 2021). Furthermore, because social cognition declines across the lifespan (Henry et al., 2013), social cognition is suggested to be a contributing factor in the increased SI/L observed in older adults, limiting the ability to establish or maintain social contacts (Cacioppo and Hawkley, 2009; de Sousa et al., 2018; Bland et al., 2022). Indeed, social cognition is a crucial factor involved in positive real-world interpersonal interactions, maintaining social relationships, and prosocial behavior (Happé et al., 1998; Pollerhoff et al., 2022; McDonald and Kanske, 2023) which, in turn, impact wellbeing and successful aging. Interestingly, theater-based interventions offer a range of social tasks (e.g., embodying characters) and interactions (e.g., discussing a performance with other participants) which have the potential to engage and promote social cognitive processes. We thus hypothesize that social cognition in older adults may be targeted and improved by theater-based interventions.

A growing body of evidence indicates that social abilities are not a uniform construct, but rather involve the complex interplay between multiple, separate social processes at both behavioral and neural levels (Blair, 2005; Shamay-Tsoory and Aharon-Peretz, 2007; Kanske et al., 2015, 2016). In particular, a distinction is made between social cognition and social affect (Kanske, 2018), the latter referring to social emotional states such as empathy (sharing or mirroring of another's emotions; De Vignemont and Singer, 2006) and compassion (a feeling of warmth or concern toward others; Klimecki et al., 2014). With respect to lifespan development, many studies suggest a decline in social cognition in older age (for review see Henry et al., 2013; Fernandes et al., 2021) and a preservation or possible enhancement of social affect (Richter and Kunzmann, 2011; Sze et al., 2012; Chen et al., 2014; Reiter et al., 2017; Stietz et al., 2021). Supporting this are two recent cross-sectional studies comparing older and younger adults by measuring empathy, compassion, and social cognition within the same sample and naturalistic task (Kanske et al., 2015; Reiter et al., 2017; Stietz et al., 2021). These studies found that younger adults significantly outperform older adults in social cognition, indicating an age-related decline in social cognitive abilities, while empathy did not differ and compassion was greater in older adults. Thus, the current evidence suggests that social cognition and social affect age differently across the adult lifespan. In particular, the weight of evidence suggests that social cognition declines in older age.

With respect to strengthening social cognitive abilities, increasing data demonstrate that social cognition is in principle plastic, with mental training interventions inducing changes at both the behavioral and neural levels (Valk et al., 2017; Trautwein et al., 2020), including in older adults (Cavallini et al., 2015; Lecce et al., 2015). Moreover, there is a growing interest in the potential for various artistic endeavors to improve social cognition, including music (McDonald et al., 2020), film (Castano, 2021), dance (Koehne et al., 2016), and literature (Dodell-Feder and Tamir, 2018). Regarding improving social cognition in the context of theater-based interventions, it has been previously argued that acting training provides a potential means of improving social cognition and empathy by requiring participants to take the perspective and embody the emotions of various characters (McDonald et al., 2022). This theoretical argument suggests that the process of character creation and performance inherent in theatrical performance actively recruits social cognition, through which repeated engagement has the potential to foster improvements by strengthening underlying brain networks. Preliminary evidence supporting these claims comes from correlational studies, which have demonstrated that both empathy and social cognition are significantly higher in amateur and trained actors (Nettle, 2006; Goldstein et al., 2009). Or, going beyond these correlational observations, social cognition and empathy have been observed to increase in children/adolescents after acting training interventions compared to other arts training, such as visual arts or music interventions (Schellenberg, 2004; Goldstein and Winner, 2012). In addition, a growing body of research is looking into the potential of theatrical interventions for autism spectrum disorders (Corbett et al., 2011, 2019; Gabriel et al., 2016) and schizophrenia (Mele et al., 2019; Tang et al., 2020) as a means of improving social skills, as well as in Parkinson's disease as an add-on therapeutic intervention for social and emotional rehabilitation (Mirabella et al., 2017).

Beyond the theoretical potential of theatrical performance, theater-based interventions are also able to directly facilitate social interactions in practice (Noice and Noice, 2021). Importantly, there is an association between the frequency and intensity of social interactions and the maintenance of social cognitive abilities (Pearlman-Avnion et al., 2018). Moreover, a restricted social circle diminishes chances to engage with the perspectives and thoughts of others, particularly those beyond one's family ties (Lecce et al., 2015), while those individuals with strong social connections have higher social cognition measures compared to individuals with a diminished social network (Lecce et al., 2015). There is additionally a link between decreased social interaction and the age-related decline in social cognition (Bailey et al., 2008). Thus, the fostering of varied and complex social interactions may have the potential to help maintain and improve social cognition by providing novel opportunities for older adults to reflect upon other people's mental states, feelings, and beliefs. Indeed, one of the most promising features of theater-based interventions is that they provide the practical opportunity for frequent social interactions as outlined in the previous section. We thus suggest here that theater-based interventions may be able to strengthen social cognitive abilities in older adults by (i) intrinsically encouraging participants to frequently take the mental perspective and embody the emotions of various characters, and (ii) facilitating complex and varied social interactions which help expand participants' social networks.

However, we note that no study has yet specifically examined changes in social cognition in older adults partaking in theater-based interventions. Moreover, it is unclear whether such changes could directly mediate reductions in SI/L or promote successful aging. Interestingly, in their scoping review of the field, Fakoya et al. (2020) identify that the evaluation of interventions to reduce SI/L with older adults should go beyond questions of mere effectiveness to also identify *mechanisms of action*, that is, the underlying factors which facilitate SI/L reductions. Improvements in social cognition via theater-based interventions may be one such mechanism. However, we note that any mediation or moderation in SI/L reduction likely involves the interplay between numerous socio- and psychometrics (e.g., gender, depression scores, personality traits, social network size, marital status, etc.). We present social cognition as one promising, illustrative example that future studies may consider. As such, a worthwhile goal of future research will be to explore the relationships between theatrical training, improvements in social cognition, and a corresponding reduction in SI/L in older adults. Importantly, any such future investigations will benefit from having an active leisure control group (e.g., pottery, yoga, music or dance interventions) which are physical and highly stimulating but lack a specific focus on engaging social cognition, in comparison to theater-based interventions with the specific goal of engaging and improving social cognitive abilities. Second, to capture the real-world impact of theater-based interventions a clear quantification of effect sizes with regard to social cognition improvement and SI/L reduction is encouraged. Finally, with respect to the implementation and limitations, a common general issue highlighted by both policy organizations (Age UK, 2015; World Health Organization, 2021) and the academic literature (Cotterell et al., 2018; Hoang et al., 2022) is the limited evidence base supporting any specific type of intervention to reduce SI/L or encourage successful aging in older adults. Similarly, as the charity Age UK (2015) discuss in their reports on the topic, the translation from academic papers to successful real-world interventions also constitutes a significant gap in practice (Jopling, 2015). Both of these issues affect theater-based interventions and future research would thus benefit from a focus on real-world applicability, as well as effectiveness and mechanism. It must also be considered whether there are issues with participation and compliance in theater-based intervention among older adults suffering from SI/L that may in turn influence the effectiveness. In a similar vein, the demographic diversity (e.g., gender, cultural background) of older populations may add further complexities to the delivery of any standardized interventions (Fakoya et al., 2020) and therefore must also be taken into account in future investigations and policy decisions related to theater-based interventions with older adults.

## Conclusion

Ultimately, when taken together, epidemiological evidence indicates that SI/L in elderly populations represents a wide-ranging and significant healthcare challenge across nations, with multiple interrelated risk factors contributing to these social experiences. Older adults impacted by SI/L are a significant concern due to the considerable risk of negative sequelae for mental health and physical health, along with the corresponding impact on wellbeing, making the development of interventions that ameliorate these issues of direct practical importance. Here, we propose that theater-based interventions with older adults are a form of social leisure activity program that may offer a means of targeting social cognition and providing complex social interactions. In doing so, such interventions have the potential to reduce SI/L, encourage social engagement, improve wellbeing, strengthen social cognition, and ultimately facilitate successful aging in older adults.

## Author contributions

BM: Writing – review & editing, Writing – original draft. AR: Writing – review & editing, Writing – original draft. PK: Writing – review & editing, Writing – original draft.
